# Transcriptome profiling revealed multiple genes and ECM-receptor interaction pathways that may be associated with breast cancer

**DOI:** 10.1186/s11658-019-0162-0

**Published:** 2019-06-06

**Authors:** Yulong Bao, Li Wang, Lin Shi, Fen Yun, Xia Liu, Yongxia Chen, Chen Chen, Yanni Ren, Yongfeng Jia

**Affiliations:** 1 College of Basic Medicine, Inner Mongolia Medical University, Hohhot, Inner Mongolia China; 2 Department of Pathology, Inner Mongolia Medical University, Hohhot, Inner Mongolia China; 3 Tumor Molecular Diagnostic Laboratory, The Inner Mongolia Cancer Hospital, Hohhot, Inner Mongolia China

**Keywords:** Breast cancer, Transcriptome, Differentially expressed genes, ECM-receptor interaction pathway

## Abstract

**Background:**

Exploration of the genes with abnormal expression during the development of breast cancer is essential to provide a deeper understanding of the mechanisms involved. Transcriptome sequencing and bioinformatics analysis of invasive ductal carcinoma and paracancerous tissues from the same patient were performed to identify the key genes and signaling pathways related to breast cancer development.

**Methods:**

Samples of breast tumor tissue and paracancerous breast tissue were obtained from 6 patients. Sequencing used the Illumina HiSeq platform. All. Only perfectly matched clean reads were mapped to the reference genome database, further analyzed and annotated based on the reference genome information. Differentially expressed genes (DEGs) were identified using the DESeq R package (1.10.1) and DEGSeq R package (1.12.0). Using KOBAS software to execute the KEGG bioinformatics analyses, enriched signaling pathways of DEGs involved in the occurrence of breast cancer were determined. Subsequently, quantitative real time PCR was used to verify the accuracy of the expression profile of key DEGs from the RNA-seq result and to explore the expression patterns of novel cancer-related genes on 8 different clinical individuals.

**Results:**

The transcriptomic sequencing results showed 937 DEGs, including 487 upregulated and 450 downregulated genes in the breast cancer specimens. Further quantitative gene expression analysis was performed and captured 252 DEGs (201 downregulated and 51 upregulated) that showed the same differential expression pattern in all libraries. Finally, 6 upregulated DEGs (CST2, DRP2, CLEC5A, SCD, KIAA1211, DTL) and 6 downregulated DEGs (STAC2, BTNL9, CA4, CD300LG, GPIHBP1 and PIGR), were confirmed in a quantitative real time PCR comparison of breast cancer and paracancerous breast tissues from 8 clinical specimens. KEGG analysis revealed various pathway changes, including 20 upregulated and 21 downregulated gene enrichment pathways. The extracellular matrix–receptor (ECM-receptor) interaction pathway was the most enriched pathway: all genes in this pathway were DEGs, including the THBS family, collagen and fibronectin. These DEGs and the ECM-receptor interaction pathway may perform important roles in breast cancer.

**Conclusion:**

Several potential breast cancer-related genes and pathways were captured, including 7 novel upregulated genes and 76 novel downregulated genes that were not found in other studies. These genes are related to cell proliferation, movement and adhesion. They may be important for research into breast cancer mechanisms, particularly CST2 and CA4. A key signaling pathway, the ECM-receptor interaction signal pathway, was also identified as possibly involved in the development of breast cancer.

**Electronic supplementary material:**

The online version of this article (10.1186/s11658-019-0162-0) contains supplementary material, which is available to authorized users.

## Background

Breast cancer is the most common malignant tumor and the fifth leading cause of cancer-related death for women in China [1]. The morbidity and mortality for breast cancer patients are higher than for any other malignant tumor, and the risk increases annually worldwide [2]. Its genesis closely related to genetic mutations and abnormal epigenetic modifications [3]. Although substantial progress has been made in studies of genetic predisposition to breast cancer, few breakthroughs have been made regarding its development mechanism [4, 5]. Studying more diverse groups of breast cancer patients or samples could provide more insight into its cellular mechanisms. Transcriptome research would not only elucidate its cellular mechanisms and/or development progress, but also provide potential diagnostic targets [6].

A variety environmental factors, including living environment, habits and chemical exposure, contribute to the tumorigenesis of breast cancer [7]. Various genetic factors also play a role, with up to 20–40% of hereditary breast cancer patients showing particular gene mutations [8]. Many genes associated with breast cancer have been annotated and analyzed.

Mutations of breast cancer 1 (BRCA1), BRCA2 and TP53 are risk factors for a high incidence (40–66%) of breast cancer occurrence. The breast cancer 1 (BRCA1) and breast cancer 2 (BRCA2) genes normally behave as tumor suppressor genes and can maintain cell proliferation and differentiation [9]. A BRCA1 mutation can be detected in 52% of breast cancer patients [10] and up to 80% have a mutation in either BRCA1 or BRCA2 [11]. Non-mutant TP53 can regulate the life cycle of cells, mediate signaling pathways and play an important role in repairing DNA, preventing tumor recurrence and metastasis [12]. Gene polymorphism of TP53 is associated with the occurrence and development of breast cancer [13]. Some other genes, such as PTEN, ataxia telangiectasia mutated (ATM) [14], check-point kinase 2 homolog (CHEK2) [15], Rad50 DNA repair protein [16], BRCA1-interacting protein C-terminal helicase 1 (BRIP1) [17] and fibroblast growth factor receptor 2 (FGFR2) [18] can also contribute to the risk of breast cancer at a low probability.

Exploration of the genes and proteins that are abnormally expressed during the development of breast cancer is essential to provide a deeper understanding of the mechanisms involved [7]. However, differences in people’s genetics background and living environments make it very hard to unequivocally identify a common cancer-related gene for the occurrence of breast cancer. It is essential that more cancer-related genes are discovered in samples from patients with different living environments.

Transcriptome sequencing and bioinformatics analysis can efficiently evaluate whole processes in one tissue globally [19]. Whole transcriptome profiling can reveal genes that are differentially expressed in related tissues (for example, breast tumor tissues and paracancerous breast tissues). Changed genes in any cancer, including breast cancer, reflect the biological diversity of cellular phenotype and physiological function and could become important research areas for elucidating molecular mechanisms. Already, many studies have found genes or proteins strongly associated with the progress and prognosis of breast cancer, including enhancer of zeste 2 (EZH2) polycomb repressive complex 2 subunit [20] and Jab1/COPS5 [21]. In addition, the nuclear receptor-interacting protein 1 (NRIP1) and the MAPK signaling pathway can regulate the development of breast cancer cells [22].

## Materials and methods

### Study patients, tissue sample preparation and collection

Histopathological breast cancer (invasive ductal carcinoma, tumor tissue) and adjacent normal tissue (paracancerous tissues, normal tissue) samples were obtained from 14 patients with pathologically confirmed breast cancer. Six of the cases were randomly selected for transcriptome sequencing, while the other eight were selected for expression pattern studies of novel breast cancer-related genes. The samples were taken in 2016 and 2017 at the Department of Pathology of the Affiliated Hospital of Inner Mongolia Medical University in Hohhot, China. This study was approved by the Ethics Committee of Inner Mongolia Medical University. After surgery, each specimen was cut into 3–8 mm^2^ pieces. The cut tissue was immediately placed in an RNA protectant (RNAlater, Sigma Aldrich). After being infiltrated by RNAlater for 12 h at 4 °C, all samples were then quickly placed in liquid nitrogen and stored at − 80 °C until needed for further processing and sequencing.

### Isolation of total RNA from samples

Total RNA extraction was performed with TRIzol (Takara) following the manufacturer’s protocol, and isolated total RNA was stored at − 80 °C until the next step. RNA degradation and contamination was monitored using 1% agarose gel electrophoresis. A NanoPhotometer spectrophotometer (Implen) and Qubit RNA Assay Kit with a Qubit 2.0 Fluorometer (Life Technologies) were respectively used to detect the purity and concentration of total RNA. An RNA Nano 6000 Assay Kit and a Bioanalyzer 2100 system (Agilent Technologies) were used to assess the integrity of total RNA.

### Library preparation for transcriptome sequencing

At least 3 μg of total RNA per sample was used. Following the manufacturer’s instructions, the NEBNext Ultra RNA Library Prep Kit (Illumina) was used to generate the 6 pairs of sequencing libraries (6 for normal tissues and 6 for tumor tissues). Random hexamer primer, M-MuLV Reverse Transcriptase (RNase H-) and DNA Polymerase I followed by RNase H were respectively used to synthesize first- and double-stranded cDNA. Any remaining overhangs were converted into blunt ends with exonuclease and polymerase. The AMPure XP system (Beckman Coulter) was used to select cDNA fragments, preferentially those that were ~ 150–200 bp in length. Three microliters of USER Enzyme was used with size-selected, adaptor-ligated cDNA at 37 °C for 15 min followed by 5 min at 95 °C, and then PCR was performed. Two pairs of cDNA libraries were constructed: one from the cDNA libraries from 6 normal tissue samples (named N1 to N6) and the other from the cDNA libraries from 6 tumor tissue samples (named T1 to T6). The Illumina HiSeq platform (Beijing Novo Gene Biological) was used for transcriptome sequencing according to the manufacturer’s instructions.

### Bioinformatics analysis

Raw (sequenced) reads were obtained first. After raw read filtering, sequencing error rate check (Q20 and Q30) and GC content profiling, the high-quality clean paired-end reads from each sample were aligned to the reference genome using TopHat version 2.0.9. The mapped genes from the reference genome were queried in databases such as UniProtKB/SwissProt and the Non-Redundant Protein Database (NRPD) with the help of BLASTX program (E-value cut-off of 1e^− 5^). The mapped read numbers in the exon and intron regions (exonic and intronic rates) and total mapping rate were analyzed independently using HTSeq version 0.6.1. The total number of mapped reads was determined and the RPKMs (reads per kilobase of exon model per million mapped reads) were calculated for each annotated gene. The software R package of DESeq was employed to capture the DEGs (differentially expressed genes) between same pair transcriptome data from the same individual and calculate the fold changes for each gene. Genes with fold changes > 2, q values < 0.01 and FDRs < 0.01 were defined as DEGs and captured for further analysis. All DEGs were enriched to the KEGG signal pathway based on a q value < 0.01 and FDR < 0.01.

The results for DEGs obtained in this study were compared to corresponding breast cancer research transcriptome information from the GEO database (especially the latest research GSE33447 and GSE109169).

### Validation and clinical experiments with quantitative real time PCR

The validation experiment was performed with the same 6 pairs of breast cancer tissue and adjacent normal tissue used for the transcriptome sequencing. The following 12 genes were selected to perform the quantitative real time PCR: pituitary tumor-transforming 1 (PTTG1), TTK protein kinase (TTK), COL10A1, CYCS, eukaryotic translation elongation factor 1 alpha 2 (EEF1A), BUB1B, CCNB1, CDC20, karyopherin alpha 2 (RAG cohort 1 importin alpha 1; KPNA2), tetraspanin 1 (TSPAN1), tetraspanin 13 (TSPAN13) and tetraspanin 15 (TSPAN15). The group includes cancer-related genes identified in previous research. Primers were designed and are listed in Additional file 1: Table S1. 18S ribosomal RNA expression was used as an internal reference. The reaction system consisted of 2 × Super Real PreMix Plus, forward primer (10 μM), reverse primer (10 μM), cDNA and 50 × ROX Reference Dye, and the volumes of RNase-Free ddH_2_O used were 0.4, 0.6, 1, 7.4 and 10 μl. The PCR amplifications were performed in triplicate wells with initial denaturation at 95 °C for 30 s, followed by 40 cycles of 95 °C for 5 s and 60 °C for 34 s.

The clinical experiment was prepared with the total RNA from the other different 8 pairs of breast cancer tissue and adjacent normal tissue. The first-strand cDNA was synthesized using a PrimeScript RT reagent Kit with gDNA Eraser (Perfect real time PCR). The expression levels of upregulated and downregulated genes that were selected as novel breast cancer-related genes were verified via quantitative real time PCR. The primers, PCR system and amplification conditions were the same as in the validation experiment. The data were analyzed using ABI 7500 HT SDS software 4.1 (Applied Biosystems). DEG expression levels were analyzed using the 2^-ΔΔCT^ analysis method.

## Results

### The sequencing and transcriptome data

The relevant parameters, including raw reads, clean reads and total mapped rate of breast cancer tissue and normal breast tissue are summarized in Table 1. Based on filtered sequenced reads, we obtained 164,352,319 clean reads in normal tissues and 166,067,405 in tumor tissues. The average Q20, Q30, exonic, intronic and total mapping rates for tumor samples were 96.18, 90.9, 80.37, 15.8 and 92.88%, respectively. All raw reads were submitted to the NCBI SRA database under the accession number PRJNA528582.

**Table 1:** The details of the transcriptome assembly result

Sample name	Raw reads	Clean reads	Clean bases	Total mapping rate (%)	Q20 (%)	Q30 (%)	Exonic	Intronic
N1	26,062,090	26,062,090	7.82G	93.37	96.91	92.34	81.18	15.28
N2	28,333,875	28,333,875	8.5G	92.49	96.97	92.61	75	20.16
N3	23,342,517	23,342,517	7.0G	93.59	97.11	92.8	74.14	20.96
N4	31,528,594	30,258,328	9.08G	86.85	96.09	90.75	89.19	7.88
N5	23,513,213	22,160,567	6.65G	90.18	95.2	88.91	86.13	10.32
N6	31,572,030	29,732,384	8.92G	89.70	95	88.59	85.46	10.98
T1	27,667,902	27,667,902	8.3G	93.19	96.88	92.33	83.1	13.85
T2	26,369,158	26,369,158	7.91G	93.89	96.84	92.3	72.62	22.07
T3	26,583,360	26,583,360	7.98G	92.58	97.02	92.73	74.98	20.07
T4	23,947,809	22,670,303	6.8G	91.81	95.49	89.41	84.2	12.96
T5	36,079,555	34,114,397	10.23G	92.74	95.39	89.25	84.71	12.06
T6	25,419,621	24,060,173	7.22G	93.06	95.46	89.39	82.59	13.8

In total, 39,649 different genes were annotated from the whole transcriptome. Within these sequences, 4685 lncRNAs, 923 miRNAs, 926 misc. RNAs, 170 rRNAs and 18,800 protein-coding genes were annotated based on the various reference databases. In total, 18,013 genes were known protein-coding genes and 787 sequences were novel genes that were not mentioned in any database. These unknown protein-coding genes may be novel genes.

### Search for known cancer-related genes in breast cancer tissue

In total, 93 different previously reported cancer-related genes were annotated in this study (Additional file 2: Table S2). This included 7 breast cancer-related genes (Table 2): caspase 8 (CASP8), cadherin 1 type 1 (CDH1), estrogen receptor 1 (ESR1), ETS variant 6 (ETV6), forkhead box A1 (FOXA1), GATA-binding protein 3 (GATA3) and neurotrophic tyrosine kinase receptor type 3 (NTRK3). The expression levels of GATA3 and ESR1, which are both the breast tumor-related genes, showed upregulation in tumor tissues compared with normal tissues. The GATA3 expression level was 15,000 in tumor tissues and 5000 in normal tissues. The expression level was ESR1 4700 in tumor tissues and 1500 in normal tissues.

**Table 2:** Breast cancer-related genes

Gene symbol	Tumor types
CASP8	Hepatocellular; oral squamous cell; breast
CDH1	Lobular breast; gastric
ESR1	Breast
ETV6	Congenital fibrosarcoma; multiple leukemia and lymphoma types including ALL; secretory breast; MDS
FOXA1	Breast; prostate
GATA3	Breast
NTRK3	Congenital fibrosarcoma; secretory breast

Of the 93 cancer-related genes, 58 were downregulated in the tumor tissue transcriptome. The WNT inhibitory factor 1 (WIF1) gene, which is related to the tumor type pleomorphic salivary gland adenoma, was the gene with the greatest downregulation (16-fold change), while a member of the ETS family of transcription factors (ETV6), which is related to non-small cell lung cancer, had the joint smallest downregulation (0.64-fold change). Only neurotrophic tyrosine kinase receptor type 3 (NTRK3; 5.76-fold downregulation) and ETV6 (0.64-fold downregulation) were related to breast cancer. Of the 35 upregulated genes, 5 were all related to breast tumor types: CASP8 (0.7-fold upregulation), CDH1 (1.21-fold upregulation), GATA3 (3-fold upregulation), ESR1 (3-fold upregulation) and FOXA1 (+ 2.89-fold upregulation).

### Validate the accuracy of the transcriptome expression results using quantitative real time PCR

**Fig. 1:**
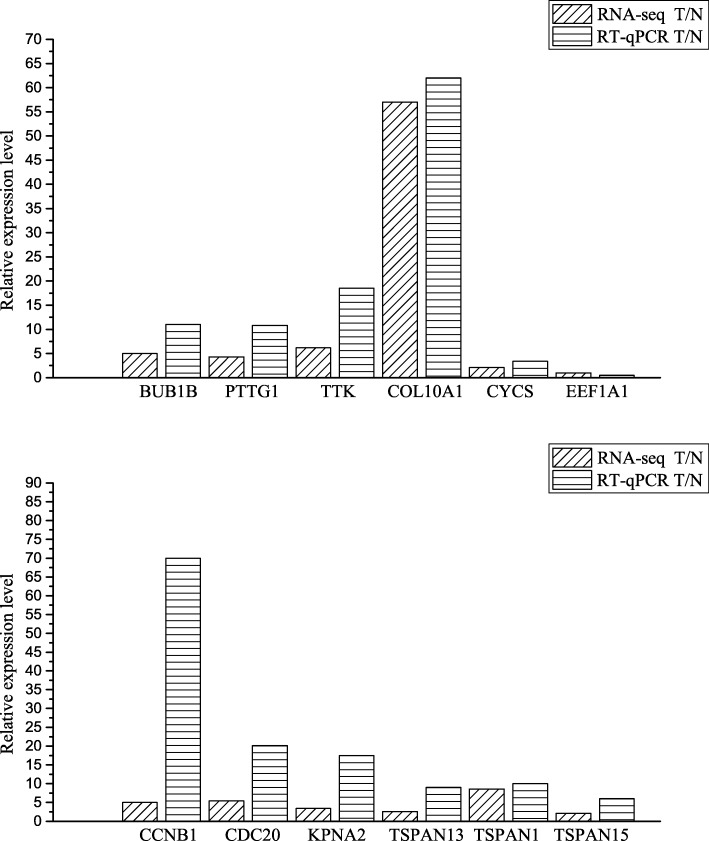
Comparison of the relative fold changes between the tumor (T) and normal (N) tissues assessed using RNA-seq and quantitative real time PCR results. Fold changes are expressed as the ratios of gene expression in the tumor tissue to that in the normal tissue, normalized to 18S rRNA. T/N indicates the gene expression in the tumor tissue normalized to that for the normal tissue

To validate the accuracy of the transcriptome expression results, we measured the expression levels of 12 randomly selected genes via quantitative real time PCR: pituitary tumor-transforming 1 (PTTG1), TTK protein kinase (TTK), COL10A1, CYCS, eukaryotic translation elongation factor 1 alpha 2 (EEF1A), BUB1B, CCNB1, CDC20, karyopherin alpha 2 (RAG cohort 1 importin alpha 1) (KPNA2), tetraspanin 1 (TSPAN1), tetraspanin 13 (TSPAN13) and tetraspanin 15 (TSPAN15). The expression patterns of these 12 genes provide evidence that the transcriptome was accurate (Fig. 1). There was a significant correlation between the two methods, with coefficients ranging from 0.91 to 0.96. This result implied that the RNA-seq result could reflect gene expression levels in the tissues.

### Identification and analysis of differentially expressed genes (DEGs)

In the tissue collections and sequencing program, the breast tumor and paracancerous tissue samples from 6 patients were treated independently. These 6 different sample pairings were sequenced, mapped, analyzed and annotated. The DESeq R package (1.10.1) and DEGSeq R package (1.12.0) were used to identify the DEGs in the different libraries from the same individual patient. Pairwise comparisons for DEG analysis were performed between the tumor tissues and paracancerous tissues in the six individual groups.

**Fig. 2:**
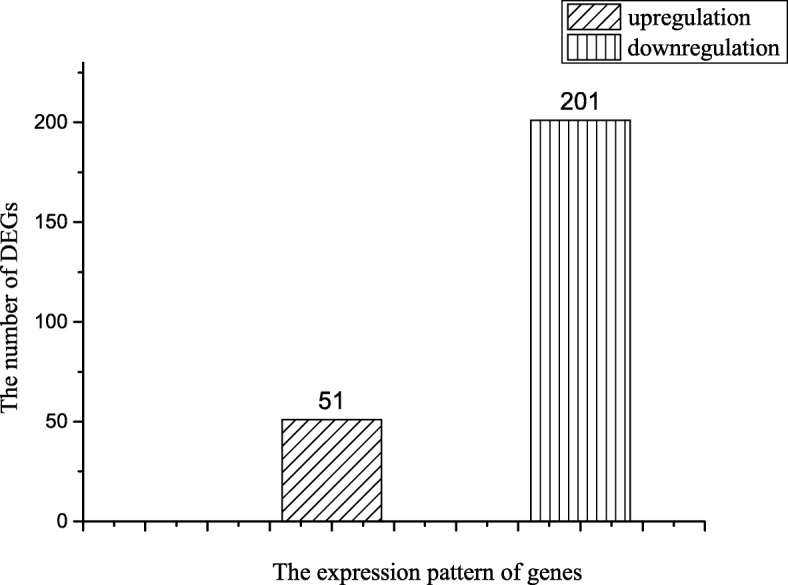
The number of differentially expressed genes (DEGs) that share the same expression patterns in all test sample pairs

Interestingly, due to individual differences, the 6 groups’ transcriptomes showed little difference in the number of DEGs (Table 3). In total, 937 DEGs (487 upregulated genes and 450 downregulated genes) were found to be differentially expressed in at least one tumor tissue compared with the paracancerous tissues within the same individual (Table 3). Further analysis shown that only 26.9% of the identified genes (252 of 937 DEGs) have a similar expression pattern among all individual groups, which indicated that the effect of individual differences must be taken into account when we define a universal cancer-related gene for breast tumors. Meanwhile, these 252 DEGs, including 51 upregulated genes and 201 downregulated genes (Fig. 2), showed the same up- or downregulation pattern in all 6 breast tumor transcriptomes with a q value < 0.01 and false discovery rate (FDR) < 0.01. Of the 51 upregulated genes, 44 were identified in the previous study (GEO result) and only 7 (CST2, DRP2, CLEC5A, SCD, KIAA1211, RP1-34B20.4, DTL) had not been studied. Of the 201 downregulated genes, 125 were identified in the previous study (GEO results) and only 76, such as cysteine rich domain 2 (STAC2), BTNL9, CA4, GPIHBP1 and PIGR, had not been studied on any cancer.

**Table 3:** The differentially expressed genes in all transcriptome groups

	Group 1	Group 2	Group 3	Group 4	Group 5	Group 6	All
Upregulated	461	418	537	556	534	474	487
Downregulated	370	336	338	316	360	411	450
Total	830	754	875	872	894	885	937

Of all the DEGs, 51 were upregulated in breast cancer tissues (Additional file 3: Table S3). Ffibronectin-1 (FN1) showed the highest expression level in the tumor tissue transcriptome: 71,967, which was 10-fold higher than that in the paracancerous tissues transcriptome. The vacuole membrane protein 1 (VMP1) gene exhibited the second highest expression level, followed by collagen triple helix repeat containing-1 (CTHRC1), inhibin beta A (INHBA), and matrix metallopeptidase 11 (MMP11). The relative expression levels of these DEGs were higher than 4000 in tumor tissues and less than 2000 in paracancerous tissues (Table 4). Of these 51 genes, 44 could be cancer-related based on the reference and previous research. Twenty genes were not mentioned in any study about breast cancer.

**Table 4:** Upregulated genes in breast cancer tissue

Gene name	log2 fold change	q value	Gene description
FN1	3.604814	1.59E-18	Fibronectin1
VMP1	1.625331	3.89E-09	Vacuole membrane protein1
CTHRC1	2.77433	2.99E-11	Collagen triple helix repeat containing 1
INHBA-AS1	0.559233	0.348753	INHBA antisense RNA1
MMP11	5.286475	7.26E-19	Matrix metallopeptidase11
FOXA1	1.699589	1.05E-06	Forkhead box A1
CST2	5.665185	2.98E-12	Cystatin SA
HIST1H2AI	5.189283	1.84E-13	Histonecluster1 H2ai
HIST1H2BF	4.235548	1.39E-09	NA
DRP2	3.339072	5.25E-10	Dystrophin-related protein 2
ASPM	2.946803	5.58E-09	Abnormal spindle microtubule assembly

More genes in the cancer transcriptome showed a downregulated expression pattern than an upregulated one. A total of 201 DEGs were downregulated (Additional file 4: Table S4). The top 10 genes with the largest differences were delta-like 1 homolog (DLK1), CA4, phospholipid phosphatase related 1 (LPPR1), adhesion G protein-coupled receptor D2 (GPR144), CD300LG, heparanase 2 (HPSE2), solute carrier family 13 (sodium-dependent dicarboxylate transporter) member 2 (SLC13A2), heparan sulfate-glucosamine 3-sulfotransferase 4 (HS3ST4), polymeric immunoglobulin receptor (PIGR), and ciliary neurotrophic factor receptor (CNTFR). These genes were downregulated by 5- to 128-fold.

The reads per kilobase of transcript per million mapped reads (RPKM values) of these genes were not less than 2000 in normal tissues but were more than 700 in tumor tissues. Two genes, PIGR and BTNL9, showed downregulation by 32-fold and 26-fold, respectively, in tumor tissues compared to normal tissues (Table 5).

**Table 5:** Downregulated genes in breast cancer tissue

Gene name	log2 fold change	q value	Gene description
DLK1	−5.55726	9.45E-17	Delta-like1homolog (Drosophila)
CA4	−5.33851	4.63E-10	Carbonic anhydrase IV
LPPR1	−3.26132	1.17E-07	Phospholipid phosphatase related1
GPR144	−6.65466	1.85E-32	Adhesion G protein-coupled receptor D2
CD300LG	−4.33055	1.26E-09	CD300 molecule like family member g
HPSE2	−4.70027	6.79E-13	Heparanase 2 (inactive)
SLC13A2	−4.26341	5.06E-16	Solute carrier family13 (sodium-dependent dicarboxylate transporter) member 2
HS3ST4	−4.99125	8.68E-16	Heparan sulfate-glucosamine3-sulfo transferase 4
PIGR	−4.83315	1.31E-28	Polymeric immune globulin receptor
CNTFR	−3.40391	9.08E-10	Ciliary neuro trophic factor receptor
BTNL9	−3.07588	4.88E-08	Butyrophilin-like9
CDH12	−3.86619	4.58E-08	Cadherin12 type 2 (N-cadherin2)
GNA11	−0.73688	0.00466	Guanine nucleotide-binding protein (Gprotein) alpha11 (Gqclass)
DLK1	−5.55726	9.45E-17	Delta-like 1 homolog (*Drosophila*)
GPIHBP1	−3.70459	4.43E-12	NA

### KEGG pathway enrichment analysis

KEGG is a database for the molecular or system biology study of gene clusters. These genes perform their functions at different levels (e.g., cell and organism levels). KOBAS software was used to test the statistical enrichment of DEGs in the KEGG pathways. A total of 937 DEGs were enriched in 219 different KEGG pathways, and 41 significant DEG-enriched KEGG pathways (21 downregulated pathways and 20 upregulated pathways) were annotated.

**Fig. 3:**
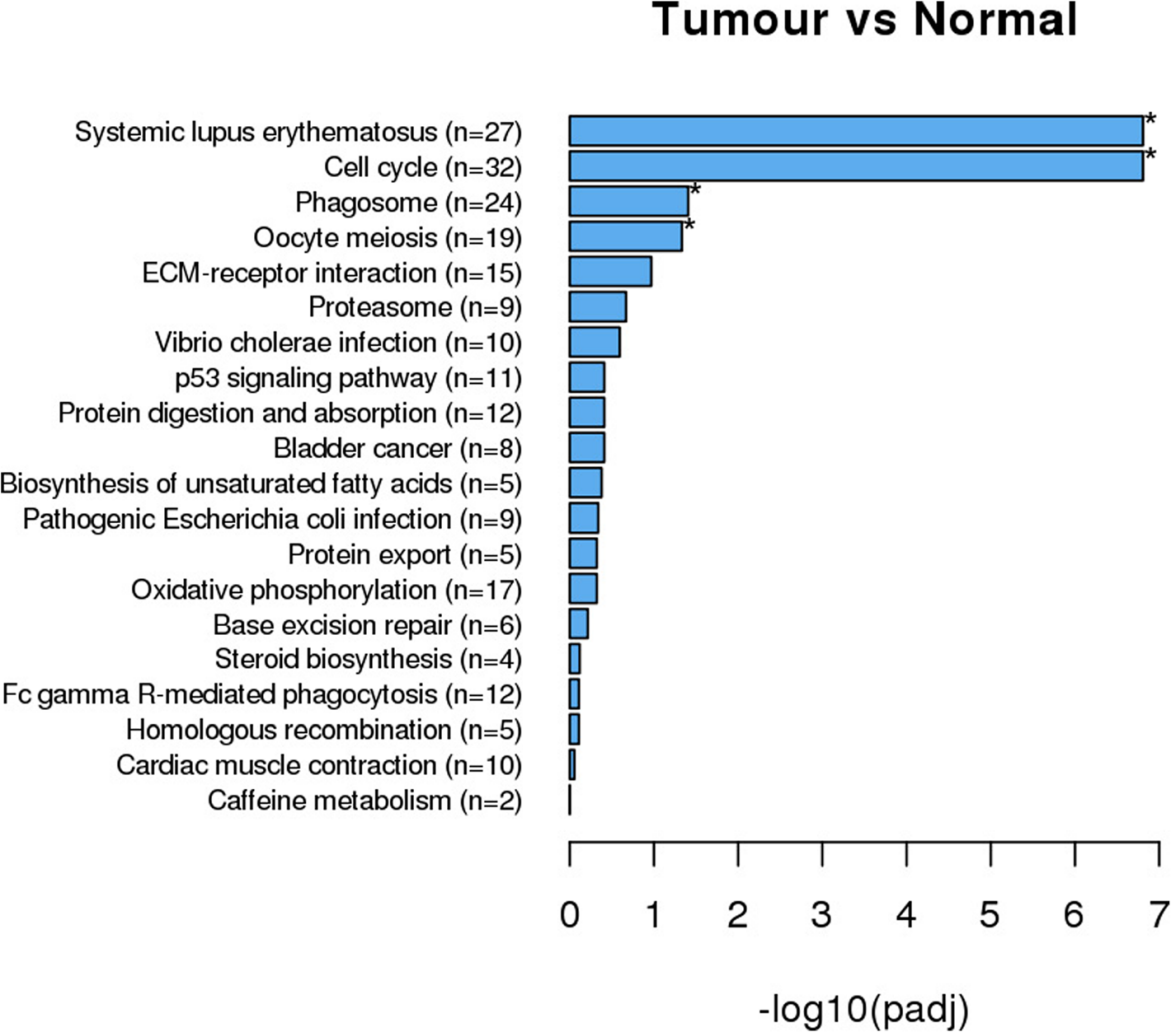
KEGG pathways significantly enriched with upregulated genes. n = the number of DEGs enriched in the pathway. X-axis represents the q value. *p < 0.05

**Fig. 4:**
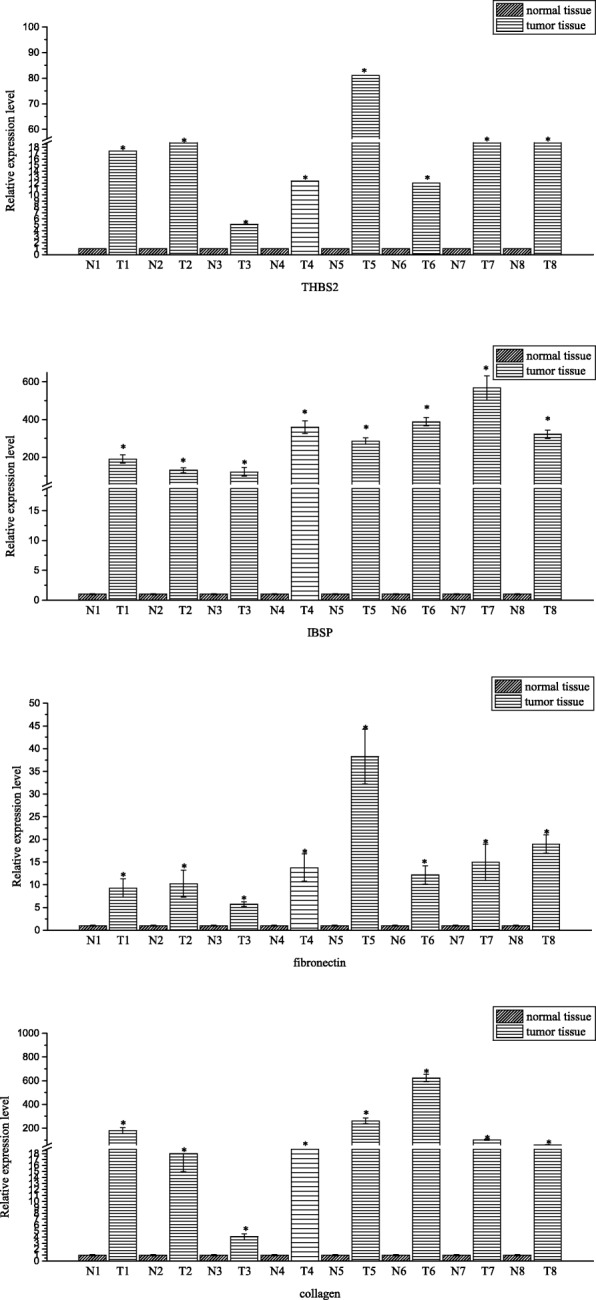
The relative expressions of THBS2, IBSP, fibronectin and collagen in normal tissues and tumor tissues assessed via quantitative real time PCR. Fold changes are expressed as the ratio of gene expression in tumor tissue to that in normal tissue, normalized to 18S rRNA. The gene expression in normal tissue is normalized to 1. *p < 0.05, **p < 0.01

Among the upregulated pathways, the extracellular matrix–receptor (ECM-receptor) interaction (22 DEGs), systemic lupus erythematosus (27 DEGs), phagosome (24 DEGs), oocyte meiosis (19 DEGs) and cell cycle (32 DEGs) pathways were significantly enriched in all 6 transcriptomes. All DEGs annotated in the ECM-receptor interaction pathway, including collagen, THBS, fibronectin and BSP, were upregulated in the tumor tissues (Figs. 3 and 4).

**Fig. 5:**
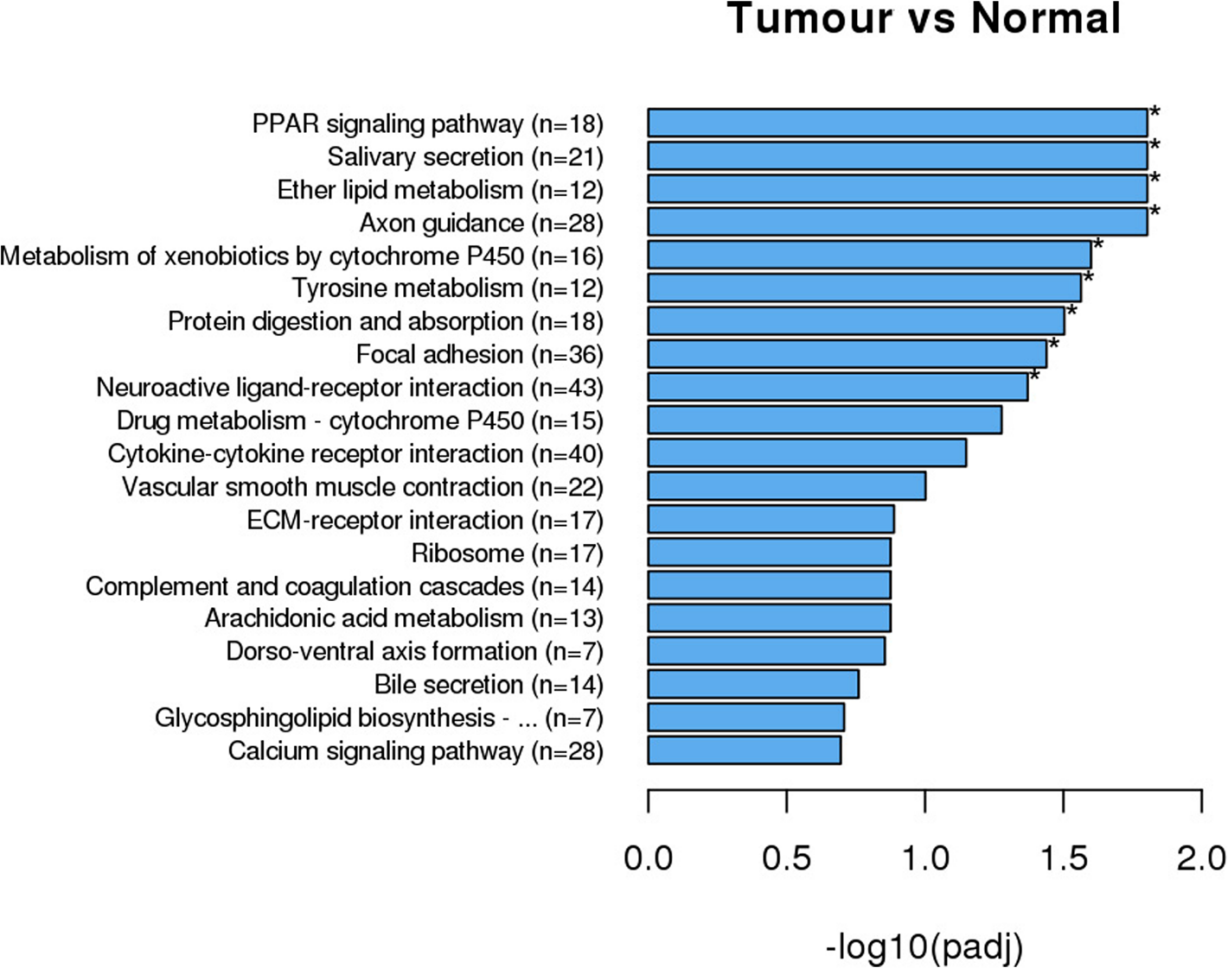
KEGG pathways significantly enriched with downregulated genes. n = the number of DEGs enriched in the pathway. X-axis represents the q value. *p < 0.05

Similarly, 9 downregulated pathways were significantly enriched: the axon guidance pathway (28 DEGs), ether lipid metabolism pathway (12 DEGs), salivary secretion pathway (21 DEGs), PPAR signaling pathway (18 DEGs), metabolism of xenobiotics by cytochrome P450 pathway (16 DEGs), tyrosine metabolism pathway (12 DEGs), protein digestion and absorption pathway (18 DEGs), focal adhesion pathway (36 DEGs) and neuroactive ligand-receptor interaction pathway (43 DEGs). The PPAR signaling pathway was annotated as a downregulated DEG enrichment pathway in all different 6 transcriptomes, and the 18 DEGs, including fatty acid binding protein 7 brain (FABP7), solute carrier family 27 (fatty acid transporter) member 6 (SLC27A6), solute carrier family 27 (fatty acid transporter) member 1 (SLC27A1) and collagen domain-containing (ADIPOQ), showed downregulation by 1.5-fold to 6.7-fold in all sequencing groups (Fig. 5).

### Search for potential cancer-related genes in DEGs from breast cancer tissue

Only genes that showed the same expression pattern in all 6 transcriptome pairs were taken into consideration. Of these 51 genes, CST2 showed the biggest expression differences between tumor tissues and paracancerous tissues (350-fold upregulation). Only ~ 1 mean relative expression level was detected in the normal tissues. Functional analysis revealed that this gene is a protein-coding gene, 748 bp in length, and located on chromosome 20. The other genes with high fold changes, dystrophin related protein 2 (DRP2) and COL10A1, were also annotated. COL10A1 showed a relative expression level of 3937 in breast tumor tissues and only 21 in paracancerous breast tissues.

Of the 201 downregulated genes, DLK1 exhibited a 128-fold downregulation in breast tumor tissues. However, the RPKM values of this gene were not very high in the transcriptomes (37 in normal tissue and 0.3 in tumor tissue). Its low expression level may mean it is not a good cancer-related gene for breast tumors. CD300LG and BTNL9, which exhibited more than 32-fold downregulation in all the tumor transcriptomes, showed a very high differential expression patterns. The expression level of CD300LG (2343 RPKM) and BTNL9 (7326 RPKM) were very high in normal tissues but very low in the tumor transcriptome (56 RPKM and 283 RPKM, respectively). The same result was observed in the expression pattern of polymeric immunoglobulin receptor (PIGR), which demonstrated a negative 32-fold change (12,789 RPKM in normal tissues and 412 RPKM in tumor tissues). These genes could be potential low expression level breast cancer-related genes.

### The clinical experimental with quantitative real time PCR

**Fig. 6:**
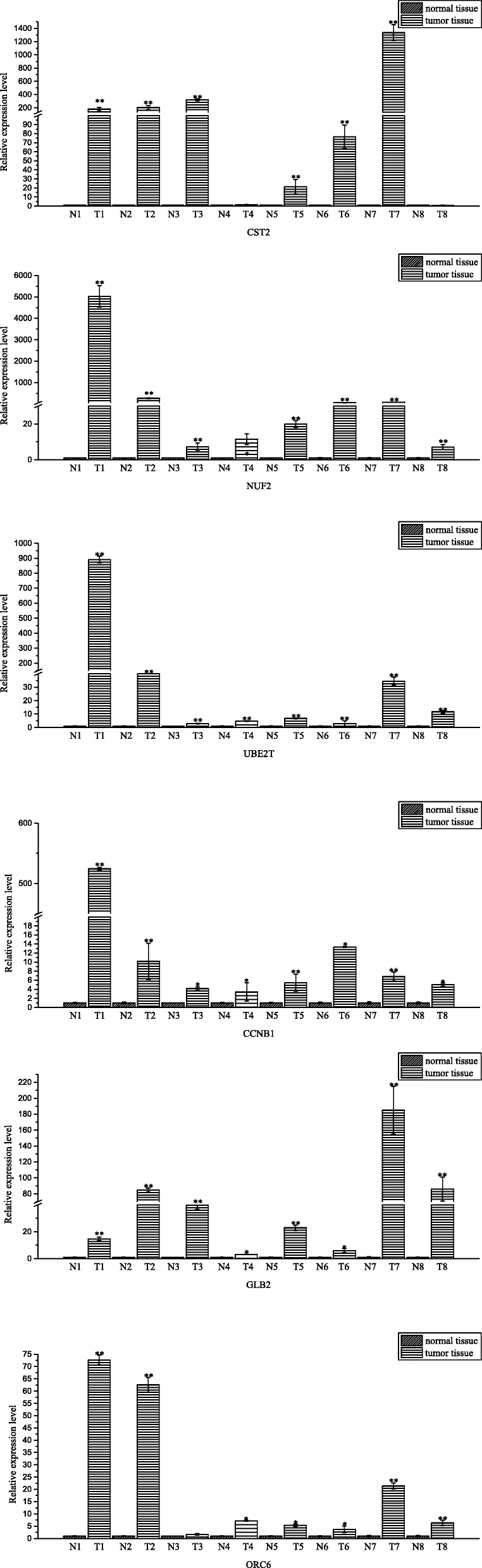
The relative expressions of CST2, GJB2, NUF2T, NUF2, ORC6 and CCNB1 in normal tissues and tumor tissues assessed via quantitative real time PCR. Fold changes are expressed as the ratio of gene expression in tumor tissue to that in normal tissue, normalized to 18S rRNA. The gene expression in normal tissue is normalized to 1. *p < 0.05, **p < 0.01

**Fig. 7:**
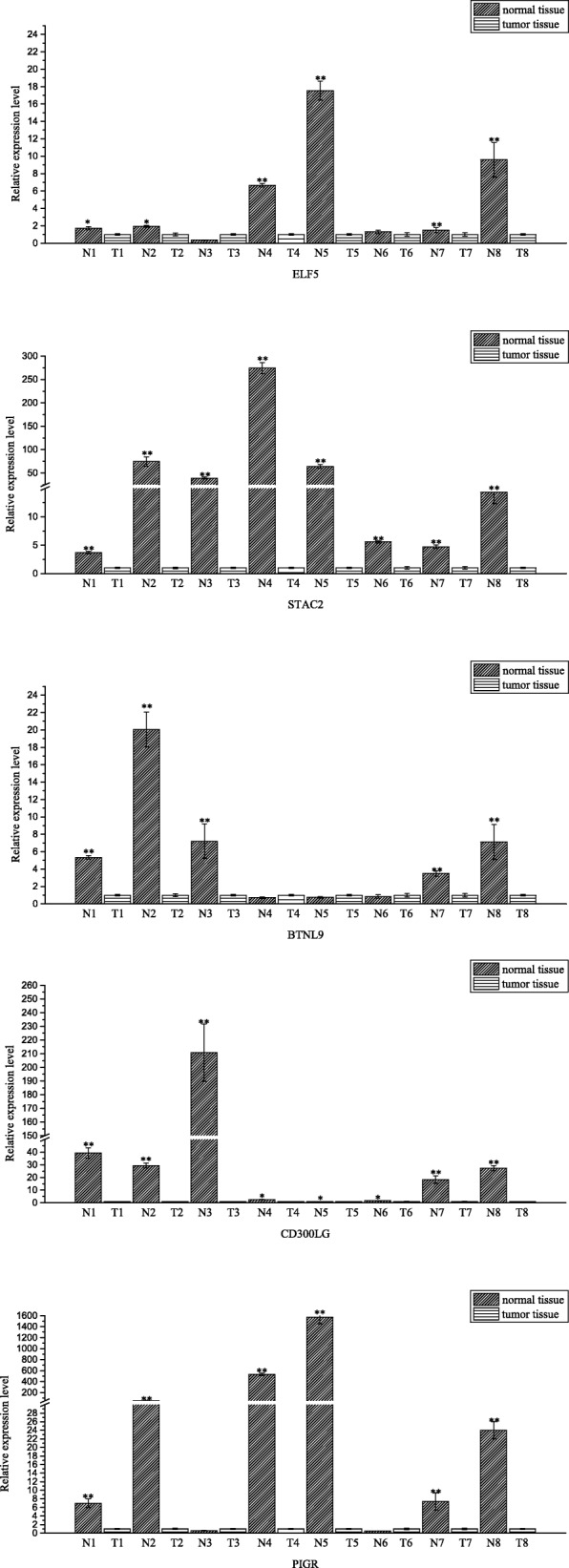
The relative expressions of BTNL9, CA4, CD300LG, ELF5, PIGR and STAC2 in normal tissues and tumor tissues assessed via quantitative real time PCR. Fold changes are expressed as the ratio of gene expression in tumor tissue to that in normal tissue, normalized to 18S rRNA. The gene expression in normal tissue is normalized to 1. *p < 0.05, **p < 0.01

To determine the clinical effects, we screened 6 high expression level and 6 low expression level genes to determine expression patterns in breast cancer tissues and adjacent tissues from 8 different patients. All quantitative real time PCR primers were designed based on the gene sequences reported in the NCBI database (Additional file 1: Table S1 primers). The results showed that the upregulated CST2, GJB2, UBE2T, NUF2, ORC6 and CCNB1 (Fig. 6), and the downregulated ELF5, cysteine-rich domain 2 (STAC2), BTNL9, CA4, CD300LG and PIGR (Fig. 7) showed the same result in different patients. This also verified the RNA-seq results. These 12 genes could be new cancer-related genes for the clinical treatment of breast cancer.

## Discussion

Using next-generation sequencing technology and quantitative real time PCR, we successfully analyzed the DEGs in breast cancer tissues from patients from Inner Mongolia in China. Since the molecular changes in breast cancer tissues are dependent on tumor type, grade, size and receptor status [23–25], we limited our study to invasive cases. Transcriptome sequencing techniques play an important role in the identification of cancer-specific genes [3, 5, 6, 19]. We sequenced the transcriptome from 6 pairs of breast cancer tissues and adjacent normal tissues and compared expressions in each pair, finding that 51 DEGs were upregulated and 201 DEGs were downregulated.

Because the gene expression patterns or the transcriptomes of cancer patients are greatly impacted by multiple factors, including living environment [26] and the severity of the disease [27], there can be considerable inter-patient variation. The DEG results in this study support the fluctuations in the number of DEGs between the breast cancer tissue and paracancerous tissue in different individuals. They also confirm that the expression levels of DEGs display significant differences between breast cancer patients.

At the same time, the statistical results of all DEGs in our study showed that each patient expressed unique DEGs (937 DEGs in total and 253 DEGs in common). The expression patterns of many DEGs in the transcriptome were not stable, which may due to the development of the disease or the genetic background of the individual [7]. This is a hindrance for researchers seeking universal cancer-related genes for breast cancer. Therefore, individual differences must be taken into account when conducting subsequent studies.

The expressions of three tetraspanin family members, TSPAN1, TSPAN13 and TSPAN15, are upregulated.They all function as transmembrane transport proteins, and TSPAN15 is also associated with the notch signaling pathway [28, 29]. TSPAN1 has been reported to regulate the progression of many human cancers, including gastric cancer, pancreatic cancer and cervical cancer [30–32]. Meanwhile, the expression of TSPAN1 was higher in ER-positive and HER2-positive breast cancer [33]. All samples in this study were collected from ER-positive patients. While the expression of TSPAN13 in prostate cancer and glioma is known to be elevated [34, 35], there is only one study on TSPAN13 in breast cancer [36]. It mentioned that TSPAN13 was upregulated in breast cancer cells. There are few studies on TSPAN15, and its effect on cancer was reported less frequently.

In our results, the expression levels of TSPAN1, TSPAN13, and TSPAN15 in breast cancer were all increased. Our TSPAN1 results are consistent with those previously reported [33], so we speculate that TSPAN13 and TSPAN15 may be potential cancer-related genes for breast cancer. This needs further study.

Our validation showed that the expression patterns of BUB1B, CCNB1, CDC20, COL10A1, CYCS, EEF1A2, gap junction protein beta 2 (GJB2), KPNA2, PTTG1, RAB31, TTK, UBE2C, ELF5 and STAC2 were the same in all individual patients. These genes have been reported as cancer-related for breast cancer [23–25, 37–45].

Previous reports [25] have shown that in invasive breast cancer, upregulated genes are related to cell proliferation and cell movement, while downregulated genes are associated with cell adhesion. Our study showed that ASPM, INHBA, NUF2, ORC6, UBE2T and PKMYT1 are associated with cell proliferation [46–52], and the expressions of these genes were also elevated in our breast cancer tissue transcriptome. The immune function-related genes CD300LG and PIGR were also detected as downregulated in breast cancer [53, 54].

In this study, 7 upregulated and 76 downregulated DEGs were captured and may be the important genes for breast cancer research. Of the 6 upregulated genes, CST2, which belongs to the cystatin superfamily and is an active cysteine protease inhibitor [55], showed a 350-fold change compared to its expression in normal tissue. The protein of this gene is found in a variety of human fluids and secretions [55], which could provide a new detection method for breast cancer. Till now, few studies have focused on CST2 in any tumor type, except to show that is responsive to the anti-growth activity of triptolide in ovarian cancer cells [56]. More study should be performed to confirm the function and character of CST2 in the development of breast cancer.

The other high expression level gene in breast tumors was DRP2, which is associated with paranoid-type schizophrenia [57]. Some study suggests a relationship between DRP2 and lung cancer [58] and brain cancer [59]. The function of this gene in breast cancer is still unknown.

Just like the CST2, GJB2, UBE2T, NUF2 and ORC6 also showed the same high expression level in breast tumors. GJB2 is implicated in the mechanisms of invasion of ductal breast carcinoma [60] and it is a prognosis marker in pancreatic cancer [61]. The downregulation of UBE2T could inhibit the progression of gastric cancer [62] and performed the same function in prostate cancer [63]. Previous study indicated that the downregulation of NUF2 could inhibit the growth of pancreatic cancer [64]. Few studies have focused on the gene function of ORC6 in breast cancer, but single-nucleotide polymorphisms (SNPs) were detected in this breast cancer-related gene [65].

We found more genes with low expression levels in tumors: 63 with at least a 10-fold change, including BTNL9, CA4, GPIHBP1 and PIGR. In total, 6 low expression level genes were confirmed using quantitative real time PCR for 6 transcriptome specimens and 8 clinical specimens.

BTNL genes show expression pattern changes in intestinal inflammation and colon cancer [66] and may be important in tumor immunity [67]. The expression and prognostic significance of PIGR, an immunoglobulin receptor, is similar, in epithelial ovarian cancer [68]. CA4, which is involved in cell proliferation, has been shown to inhibit cell proliferation, invasion and metastasis and was downregulated in our results [69]. The glycosylphosphatidylinositol high-density lipoprotein binding protein 1 (GPIHBP1) acts to chaperone secreted LPL and interact in fatty acids and breast cancer [70]. The role of GPIHBP1 has yet to be studied in cancer.

To the best of our knowledge, the function of these genes in breast cancer has not received much coverage. More study should be performed to explore the role of these genes. An expression pattern like the one we found for these genes may imply a high risk of breast cancer.

The KEGG pathway annotation showed that all DEGs were significantly enriched in 20 pathways, including the ECM-receptor interaction pathway and the protein digestion and absorption pathway, suggesting that there are many DEGs and signaling pathways involved in breast cancer. This is also a major reason why breast cancer is so difficult to cure. ECM-receptor interaction pathways were the most upregulated gene-enriched signaling pathways. They play an important role in the process of tumor shedding, adhesion, degradation, movement and hyperplasia. The role of ECM in other cancers has been proved. ECM is upregulated in prostate cancer tissue [71] and participates in the process of tumor invasion and metastasis in gastric cancer [72]. The ECM in colorectal cancer could promote the development of epithelial–mesenchymal transition (EMT) in cancer cells [73]. Glioblastoma is the deadliest adult brain tumor. It shows pathological features of abnormal neovascularization and diffuse infiltration of tumor cells. The interactions between the ECM and the glioblastoma microenvironment are important in this progression [74].

During tumor metastasis, tumor cells pass through the ECM, and the tumor suppressor nischarin may prevent cancer cell migration by interacting with many proteins [75]. Some studies have revealed that nischarin can prevent the migration and invasion of breast cancer cells by changing the expression patterns of key adhesive proteins [76]. The expression of nischarin could reduce the ability of cells to attach to the ECM, which would lead to a decrease in invadopodium-mediated matrix degradation [77].

Invasive metastasis is a unique biological feature of malignant tumors. The high expression level of ECM proteins or genes in breast tumor tissues may provide new ideas for cancer treatment. We think that these genes and pathways may be potential markers for breast cancer, but the mechanisms of tumorigenesis and development need to be verified in further experiments.

## Additional files

Additional file 1: **Table S1.** primers. Primers for all the differentially expressed genes for quantitative real time PCR analysis. (XLSX 11 kb)

Additional file 2: **Table S2.** Tumor type-related genes. (XLSX 42 kb)

Additional file 3: **Table S3.** The 51 upregulated genes in breast cancer tissues. (XLSX 18 kb)

Additional file 4: **Table S4.** The 201 downregulated genes in breast cancer tissues. (XLSX 43 kb)

## Data Availability

All data generated or analyzed during this study are included in this published article and its supplementary information files.

## Abbreviations

BLAST: Basic Local Alignment Search Tool
DEGs: Differentially expressed genes
ECM: Extracellular matrix
KEGG: Kyoto Encyclopedia of Gene and Genomes
